# Are infant mortality rates increasing in England? The effect of extreme prematurity and early neonatal deaths

**DOI:** 10.1093/pubmed/fdaa025

**Published:** 2020-03-02

**Authors:** Selina Nath, Pia Hardelid, Ania Zylbersztejn

**Affiliations:** Population, Policy and Practice Research & Teaching Department, UCL Great Ormond Street Institute of Child Health, 30 Guilford Street, London WC1N 1EH, UK

**Keywords:** Infant mortality, perinatal mortality, stillbirth, inequality, trends

## Abstract

**Background:**

Infant mortality has been rising in England since 2014. We examined potential drivers of these trends.

**Methods:**

We used aggregate data on all live births, stillbirths and linked infant deaths in England in 2006–2016 from the Office for National Statistics. We compared trends in infant mortality rates overall, excluding births at <24 weeks of gestation, by quintile of SES and gestational age.

**Results:**

Infant mortality decreased from 4.78 deaths/1000 live births in 2006 to 3.54/1000 in 2014 (annual decrease of 0.15/1000) and increased to 3.67/1000 in 2016 (annual increase of 0.07/1000). This rise was driven by increases in deaths at 0–6 days of life. After excluding infants born at <24 weeks of gestation, infant mortality continued to decrease after 2014. The risk of infant death was 94% higher in the most versus least deprived SES quintile, which reduced to a 55% higher risk after adjusting for gestational age.

**Conclusions:**

The observed increase in infant mortality rates since 2014 is wholly explained by an increasing number of deaths at 0–6 days of age among babies born at <24 weeks of gestation. Policies focused on improving maternal health to reduce preterm birth could substantially reduce the socio-economic gap in infant survival.

## Introduction

Infant mortality is a key indicator of population health. Despite large declines in infant mortality rates during the twentieth- and twenty-first centuries, the UK has higher rates of and a wider socio-economic gap in infant mortality compared to many other high-income countries with universal healthcare systems.^1^^,^^2^ Of concern are the recent reports of a rise in infant mortality rates across England from 3.6 deaths per 1000 live births in 2014 to 3.8/1000 in 2016.^3^ Further, the disparity in infant survival between the richest and poorest areas in England may be increasing.^4^^,^^5^

The interpretation of infant mortality rates is complicated by the differences in legal requirements for registration of still- and live births, particularly for extremely premature births (at <24 weeks of gestation, the group with the lowest survival rates) for which clinical decision-making may be most variable.^6^^,^^7^ All births showing signs of life are required to be registered as live births, while babies born with no signs of life are legally required to be registered as stillbirths only after 24 weeks of gestation. A wide regional variation in registration practices for extremely premature births and subsequent deaths as either infant deaths or foetal losses has contributed to the regional differences in infant mortality rates in England.^8^ Exclusion of live births at <24 weeks of gestation from calculation of infant mortality rates is recommended to ensure comparability of rates across time and place.^9^^,^^10^ In addition, risk factors for stillbirth are similar to those for infant mortality.^11^ Therefore, the perinatal mortality rate (calculated as the number of stillbirths and deaths in the first week of life per 1000 total births) could provide a more comprehensive picture of trends in early-life survival than infant mortality.

The NHS England has proposed a goal to halve rates of stillbirths and neonatal mortality (deaths at age 0–27 days) by 2025.^12^ To monitor progress towards this goal, we require a better understanding of the drivers of trends in infant mortality over time and by socio-economic status, enabled by robust analyses of mortality statistics. This will allow identification of clear target areas and subpopulations for preventive interventions.^1^^,^^13^

We examined whether the observed increases in infant mortality in England are real or a data artefact. In addition, we determined the contribution of premature birth to trends in socio-economic inequalities in stillbirth, perinatal and infant mortality rates.

## Methods

### Data and participants

A bespoke aggregate data extract was obtained from the Office for National Statistics (ONS). This included all live births, stillbirths and linked infant deaths in England between 2006 and 2016, tabulated by year of birth, gestational age category, Index of Multiple Deprivation (IMD) decile and age at death. Data are derived from ONS birth registrations, NHS birth notifications and ONS death registrations which are collected, maintained and routinely linked by ONS for publication of national statistics.

### Outcomes

Our primary outcome was the ‘infant mortality rate per 1000 live births’ based on ONS’s definition:^14^

(1) \begin{equation*} \mathrm{Infant}\ \mathrm{mortality}\ \mathrm{rate}=\frac{Deaths\ aged\ 0-364\ days}{Live\ Births}\times 1000 \end{equation*}

The infant mortality rate was broken down by age at death as early neonatal (0–6 days), late neonatal (7–27 days) and postneonatal mortality (28–364 days). Our secondary outcomes were the *stillbirth rate per 1000 total births* and the *perinatal mortality rate per 1000 total births.*^14^ All outcomes are described in detail in Supplementary data, Supplement S1.

### Exposures

The primary exposure was *socio-economic status*, measured using quintiles of 2015 IMD scores, the official measure of relative area-level deprivation in England. IMD scores were calculated for small areas covering 200–1400 households; neighbourhoods were then ranked and grouped into quintiles.^15^ IMD quintiles were then allocated to births according to the mother’s postcode at birth. The secondary exposure of interest was *gestational age* categorized as <24, 24–27, 28–31, 32–36, 37–41 and 42+ weeks.

### Statistical analyses

#### Trends over time

We derived counts and proportions of live births, stillbirths and early neonatal, late neonatal and postneonatal deaths by year of birth, IMD quintile and gestational age. We calculated mortality rates over time and by IMD quintile and gestational age with 95% confidence intervals (CI). Interrupted time series analysis (ITSA) was used to estimate trends in mortality rates before and after 2014 to assess the magnitude of change (see Supplementary data, Supplement S1 for description of ITSA model).^16^

To minimize potential bias due to variation in registration practices over time and across local areas, we excluded births with gestational age <24 weeks or with missing gestational age from all subsequent analyses. We then recalculated mortality rates by year of birth and used ITSA to compare trends before and after 2014 in births at or after 24 weeks of gestation.

#### Socio-economic inequalities

We compared mortality rates over time in the first quintile (Q1, most deprived) and the fifth quintile (Q5, least deprived). We calculated the proportion of live and stillbirths that were preterm (24–36 weeks) in Q1 and Q5.

To quantify socio-economic inequalities in mortality by age at death, we fitted log-binomial regression models with year of birth (as a continuous variable) and quintile of IMD score to calculate risk ratios (RR) for risk of death in each IMD quintile relative to the least deprived quintile (model 1). We then adjusted for gestational age to determine the contribution of prematurity to socio-economic inequalities in infant mortality (model 2). In the final step, we added an interaction term between year and quintile of IMD to investigate if inequalities in infant mortality have changed over time (model 3). We compared the models with and without the interaction term using the Akaike information criterion (AIC); a smaller AIC value was interpreted as a statistically significant improvement in model fit.

Analyses were performed in Excel and Stata/MP V.15.

### Sensitivity analyses

We derived the proportion of live births and early neonatal deaths at <24 weeks, overall and in the most and the least deprived IMD quintiles, to determine whether registration practices for infants born at <24 weeks of gestation have changed over time and by socio-economic status. We explored trends in the proportion of births and early neonatal deaths with missing gestational age and compared mortality rates overall, excluding births with missing gestational age, excluding births at <24 weeks of gestation and applying both criteria together to assess potential biases due to missing data.

**Table 1:** Descriptive statistics of study population: counts (%)

	*Live births*	*Stillbirths*	*Infant deaths*				*Perinatal deaths (stillbirths and early neonatal deaths)*
			*Early neonatal 0–6 days*	*Late neonatal 7–27 days*	*Post neonatal 28–364 days*	*Total 0–364 days*	
**Total**	7 343 432	35 823	16 390	4815	8863	30 068	52 213
**Year of birth**							
2006	635 122 (8.6%)	3422 (9.6%)	1648 (10%)	520 (11%)	866 (9.8%)	3034 (10%)	5070 (9.7%)
2007	654 500 (8.9%)	3352 (9.4%)	1636 (10%)	467 (9.7%)	978 (11%)	3081 (10%)	4988 (9.6%)
2008	672 373 (9.2%)	3378 (9.4%)	1614 (9.8%)	487 (10%)	938 (11%)	3039 (10%)	4992 (9.6%)
2009	670 627 (9.1%)	3431 (9.6%)	1549 (9.5%)	474 (9.8%)	882 (10%)	2905 (9.7%)	4980 (9.5%)
2010	682 405 (9.3%)	3456 (9.6%)	1534 (9.4%)	435 (9%)	876 (9.9%)	2845 (9.5%)	4990 (9.6%)
2011	683 900 (9.3%)	3574 (10%)	1539 (9.4%)	471 (9.8%)	821 (9.3%)	2831 (9.4%)	5113 (9.8%)
2012	693 862 (9.4%)	3345 (9.3%)	1454 (8.9%)	428 (8.9%)	801 (9%)	2683 (8.9%)	4799 (9.2%)
2013	664 157 (9%)	3068 (8.6%)	1359 (8.3%)	431 (9%)	713 (8%)	2503 (8.3%)	4427 (8.5%)
2014	660 908 (9%)	3023 (8.4%)	1297 (7.9%)	367 (7.6%)	675 (7.6%)	2339 (7.8%)	4320 (8.3%)
2015	662 959 (9%)	2912 (8.1%)	1339 (8.2%)	366 (7.6%)	660 (7.4%)	2365 (7.9%)	4251 (8.1%)
2016	662 619 (9%)	2862 (8%)	1421 (8.7%)	369 (7.7%)	653 (7.4%)	2443 (8.1%)	4283 (8.2%)
**IMD quintile**							
Q1: most deprived	1 994 140 (27%)	12 080 (34%)	5705 (35%)	1749 (36%)	3533 (40%)	10 987 (37%)	17 785 (34%)
Q2	1 646 979 (22%)	8429 (24%)	3858 (24%)	1144 (24%)	2154 (24%)	7156 (24%)	12 287 (24%)
Q3	1 370 409 (19%)	6268 (17%)	2746 (17%)	786 (16%)	1340 (15%)	4872 (16%)	9014 (17%)
Q4	1 222 241 (17%)	4954 (14%)	2199 (13%)	630 (13%)	1017 (11%)	3846 (13%)	7153 (14%)
Q5: least deprived	1 109 663 (15%)	4092 (11%)	1882 (11%)	506 (11%)	819 (9.2%)	3207 (11%)	5974 (11%)
**Gestational age (weeks)**							
**<**24	7621 (0.1%)	^a^	6030 (37%)	348 (7.2%)	253 (2.9%)	6631 (22%)	6030 (12%)
24–27	25 277 (0.34%)	9097 (25%)	3205 (20%)	1423 (30%)	1440 (16%)	6068 (20%)	12 302 (24%)
28–31	59 451 (0.81%)	5956 (17%)	1453 (8.9%)	557 (12%)	690 (7.8%)	2700 (9%)	7409 (14%)
32–36	448 354 (6.1%)	8725 (24%)	1803 (11%)	677 (14%)	1419 (16%)	3899 (13%)	10 528 (20%)
37–41	6 489 569 (88%)	11 578 (32%)	3209 (20%)	1712 (36%)	4841 (55%)	9762 (32%)	14 787 (28%)
42+	262 877 (3.6%)	351 (0.98%)	167 (1%)	48 (1%)	134 (1.5%)	349 (1.2%)	518 (0.99%)
Missing	50 283 (0.68%)	116 (0.32%)	523 (3.2%)	50 (1%)	86 (0.97%)	659 (2.2%)	639 (1.2%)

^a^Stillbirths are defined as foetal deaths after 24 weeks of pregnancy gestation; therefore, stillbirths are not reported for gestational age <24 weeks.

## Results

### Study population

In 2006–2016, 7 323 432 live births, 35 823 stillbirths and 30 068 infant deaths were recorded in England. 540 703 babies (7.4%) in the cohort were born prematurely (at < 37 weeks of gestation, Table 1). 55% of infant deaths occurred in the first week of life and a further 16% at 7–27 days after birth. Births and infant deaths were unequally distributed by IMD quintile: 27% of live births, 34% of stillbirths and 37% of infant deaths affected families living in the most deprived areas (Q1), compared to 15%, 11% and 11%, respectively, in the least deprived areas (Q5, Table 1).

### Trends in mortality rates

**Fig. 1:**
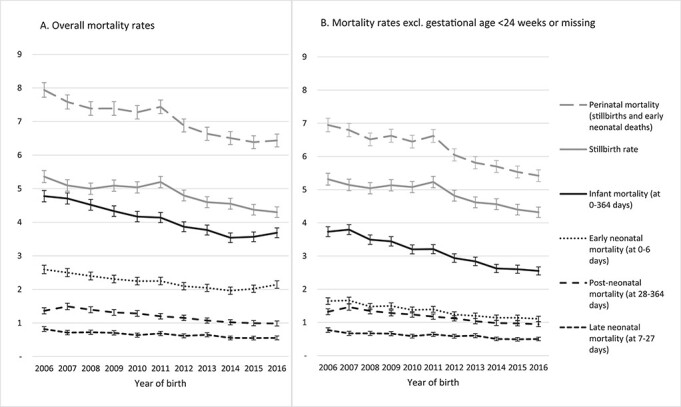
Mortality rates (95% confidence intervals) per 1000 infants during 2006–2016 overall (**A**) and excluding births and deaths at <24 weeks of gestation or with missing gestational age (**B**). All infant mortality rates were based on denominators of live births. Perinatal mortality and stillbirth rates were based on denominators of total (live and still-) births.

The infant mortality rate decreased from 4.78 deaths per 1000 live births in 2006 to 3.54/1000 live births in 2014 (an annual decrease of 0.15/1000, Fig. 1a, Supplementary Table S1) and increased to 3.57/1000 in 2015 and 3.67/1000 in 2016 (an annual increase of 0.07/1000). This increase was driven by increases in early neonatal mortality rates (an annual decrease of 0.08/1000 live births in 2006–2013, followed by an annual increase of 0.09/1000 since 2014). In contrast, late neonatal mortality has remained constant after 2014, and postneonatal mortality decreased by 0.02/1000 live births. Stillbirth rates have been decreasing since 2011 (from 5.20 stillbirths per 1000 total births in 2011 to 4.30/1000 total births in 2016). Perinatal mortality reduced from 7.94/1000 total births in 2006 to 6.44/1000 in 2016.

To minimize bias due to variation in registration practices, we excluded the 0.7% of live births and 0.3% of stillbirths with missing data and 0.1% live births at <24 weeks of gestation from all subsequent analyses. Infant mortality rates after excluding births at <24 weeks of gestation decreased over time both before and after 2014: we found statistically significant annual decreases of 0.14/1000 live births before 2014 and 0.04/1000 live births since 2014 (Fig. 1b, Supplementary Table S1).

### Socio-economic differences in mortality rates

**Fig. 2:**
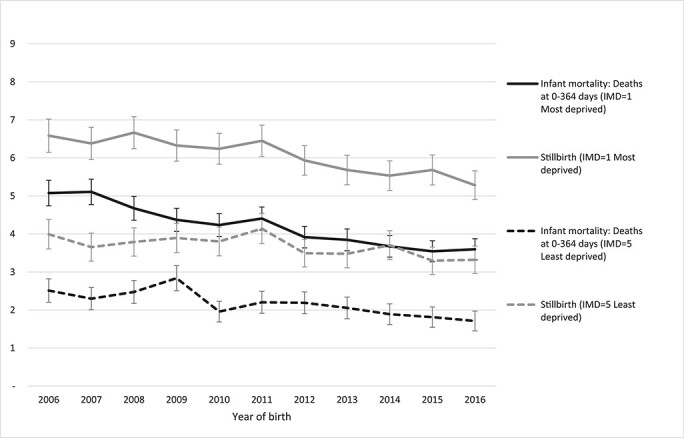
Infant mortality and stillbirth rates (95% confidence intervals) per 1000 live births or total births (for stillbirth rates) during 2006–2016, by IMD comparing IMD = 1 (most deprived) and IMD = 5 (least deprived) (excluding infants born at gestational age < 24 weeks).

The infant mortality rate in 2006–2016 was 2.89/1000 live births among children in the least deprived quintile (Q5) compared to 5.51/1000 in the most deprived quintile (Q1, Supplementary Table S2). The proportion of infants born preterm was higher in the most compared to the least deprived quintile (8.1% versus 6.6%). The risk of infant death increased with deprivation: children in Q2 had 52% higher risk of death in infancy, and children in Q1 had 94% higher risk of death compared to children in the least deprived quintile (Q5, Table 2). After accounting for gestational age, the risk of infant mortality was 31% and 55% higher in Q2 and Q1, respectively, compared to the least deprived quintile. Infant mortality has been declining over time in the most and least deprived IMD quintiles (Fig. 2).

**Table 2:** Unadjusted and adjusted risk ratios with 95% confidence intervals from log-binomial models for infant and perinatal mortality rates by year and IMD quintile in England, adjusted for gestational age

	*Infant mortality death at 0–364 days*		*Perinatal mortality^b^*		*Late neonatal death at 7–27 days*		*Postneonatal death at 28–364 days*	
	*Model 1*	*Model 2*	*Model 1*	*Model 2*	*Model 1*	*Model 2*	*Model 1*	*Model 2*
Year of birth^a^	0.96 (0.95–0.96)	0.96 (0.96–0.97)	0.97 (0.97–0.98)	0.98 (0.98–0.98)	0.96 (0.95–0.97)	0.96 (0.96–0.97)	0.96 (0.95–0.96)	0.96 (0.95–0.97)
IMD quintile								
Q1: most deprived	1.94 (1.85–2.03)	1.55 (1.49–1.62)	1.64 (1.59–1.70)	1.26 (1.22–1.30)	1.93 (1.74–2.14)	1.53 (1.38–1.70)	2.38 (2.20–2.57)	2.08 (1.92–2.24)
Q2	1.52 (1.45–1.60)	1.31 (1.26–1.38)	1.38 (1.34–1.43)	1.16 (1.13–1.20)	1.53 (1.37–1.71)	1.31 (1.18–1.46)	1.77 (1.63–1.92)	1.62 (1.49–1.76)
Q3	1.25 (1.18–1.31)	1.16 (1.11–1.22)	1.23 (1.19–1.27)	1.12 (1.09–1.16)	1.24 (1.11–1.40)	1.15 (1.03–1.29)	1.32 (1.21–1.44)	1.27 (1.16–1.38)
Q4	1.12 (1.06–1.18)	1.06 (1.01–1.12)	1.10 (1.06–1.14)	1.04 (1.00–1.07)	1.14 (1.01–1.29)	1.08 (0.96–1.21)	1.12 (1.02–1.23)	1.09 (0.99–1.18)
Q5: least deprived	Reference	Reference	Reference	Reference	Reference	Reference	Reference	Reference
Gestational age (weeks)								
24–27		153.04 (148.57–157.64)		153.86 (150.58–157.21)		206.09 (192.26–220.91)		71.98 (67.95–76.25)
28–31		29.43 (28.23–30.69)		49.13 (47.83–50.47)		34.68 (31.53–38.15)		14.95 (13.81–16.19)
32–36		5.73 (5.52–5.94)		10.08 (9.83–10.33)		5.67 (5.19–6.20)		4.17 (3.93–4.42)
37–41		Reference		Reference		Reference		Reference
42 or over		0.88 (0.78–0.97)		0.86 (0.79–0.94)		0.69 (0.51–0.91)		0.68 (0.57–0.81)

^a^Year of birth has been centred at 2006.

^b^Perinatal mortality rates are based on the total births.

Model 1 included year of birth and IMD quintile. Model 2 was further adjusted for gestational age.

The perinatal mortality rate was 4.75/1000 total births in the least deprived quintile (Q5) compared to 7.80/1000 total births in the most deprived quintile (Q1) (Supplementary Table S2). The risk of perinatal death was 38% higher in Q2 and 64% higher in Q1 (Table 2) compared to Q5. After accounting for gestational age, the risk of perinatal mortality was 16% and 26% higher in Q2 and Q1, respectively, compared to the least deprived quintile. Results for stillbirth rates and early neonatal mortality closely matched results for perinatal mortality (Supplementary Table S3).

The largest socio-economic inequalities were observed for postneonatal mortality. At 28–364 days, the mortality rate was 0.74/1000 live births in the least deprived quintile compared to 1.77/1000 in the most deprived quintile (Q1) (Supplementary Table S2). The risk of death was 77% higher in Q2 and nearly 2.4 times higher in Q1 compared to the least deprived areas. The relative risks did not change substantially after accounting for gestational age (Table 2).

We did not find statistically significant differences in the rate of mortality decline between the deprivation quintiles for any outcome, as an effect modification term between year and IMD quintile did not significantly improve the fit of the models.

### Sensitivity analyses

The proportion of live births at <24 weeks remained constant over time (Supplementary Table S4), and a higher proportion of infants were born at <24 weeks of gestation in most deprived areas compared to the least deprived areas (0.14% of live births versus 0.08%, Supplementary Table S5). There has been a steady increase in the proportion of early neonatal deaths in infants born at <24 weeks of gestation, from 35% in 2006 to 47% in 2016 (Supplementary Table S4). The proportion of early neonatal deaths in infants born at <24 weeks of gestation has been increasing over time in both the highest and lowest IMD quintiles and was comparable (39.3 versus 37.4% over the whole study period).

The proportion of live births with missing gestational age decreased from 1% in 2006–2010 to 0.3–0.4% thereafter (Supplementary Table S4). For deaths, gestational age was predominantly missing for early neonatal deaths, resulting in underreported early neonatal mortality rates based on births with complete gestational age compared to using all data (2.18 deaths per 1000 live births versus 2.23/1000, respectively, Supplementary Table S6). The proportion of early neonatal deaths with missing data on gestational age declined over time but increased from 2% in 2012 to 3.4% in 2016. Excluding births and deaths with missing data did not substantially change the trends in early neonatal mortality over time (Supplementary Figure S1).

## Discussion

### Main finding of this study

The increase in infant mortality rates since 2014 in England has been driven by increases in early neonatal deaths in infants born at <24 weeks of gestation. In contrast, infant mortality rates for children born at ≥24 weeks of gestation have been decreasing since 2006. Socio-economic inequalities in infant mortality remained constant over time. Compared to the least deprived IMD quintile, infants from the most deprived quintile had 94% higher risk of infant mortality and 64% higher risk of perinatal mortality. After accounting for gestational age, inequalities in perinatal mortality were still present but substantially reduced, but the risk of postneonatal mortality remained twice as high in the most compared to the least deprived areas.

### What is already known on this topic

Observed increases in infant mortality have been associated with rising child poverty, leading to a number of calls for the government and policymakers to take urgent action.^4^^,^^5^^,^^17^ Infant mortality rates can be influenced by variation over time and place in registration of stillbirths and extremely premature live births.^9^ Thus, exclusion of births at <24 weeks has been recommended to ensure comparability of calculated rates. A recent report from Maternal, Newborn and Infant Clinical Outcome Review Programme in the UK (run by MBRRACE-UK) applied these exclusions and showed no evidence of the increases in neonatal mortality or stillbirth rates in 2013–2017 in the UK.^18^

### What this study adds

The increases in infant mortality rates since 2014 in England were driven by increases in early neonatal deaths in infants born at <24 weeks of gestation. Infant mortality rates for children born at or after 24 weeks of gestation have been decreasing since 2006. According to routinely published ONS data, trends in early neonatal mortality were primarily driven by increases in deaths on day 0 of life (which increased from 1.33/1000 live births in 2014 to 1.60/1000 live births in 2017 for England and Wales). In contrast, mortality at 1–6 days of life showed a slow decline (from 0.65/1000 live births in 2014 to 0.58/1000 live births in 2017).^19^ These early deaths are likely to reflect increasing numbers of live births at <23 weeks of gestation (376 in 2014, 427 in 2015 to 545 in 2016), 97% of whom died in the first week of life.^20–22^ The increase in live births occurring at <23 weeks may be due to changes in the management of high-risk pregnancies^23^ and availability of more advanced specialized care for extremely preterm infants born at <24 weeks of gestation.^24^^,^^25^ To reflect these changes, the British Association of Perinatal Medicine has published an updated guidance for perinatal management of births at <27 weeks in October 2019.^26^ These recommendations could affect trends in infant mortality rates if greater numbers of babies born at <24 weeks of gestation have resuscitation attempted.

Healthcare professionals may be registering pregnancy loss at <24 weeks as live births followed by early neonatal deaths as this impacts on eligibility for maternity and paternity leave and maternity pay which are similar for stillbirth and early neonatal death.^27^ Parents who experience late pregnancy loss before 24 weeks are not entitled to any state support. Differences in registration practices could affect observed socio-economic inequalities in early neonatal mortality, since these early deaths disproportionately affect families living in the most deprived areas who would benefit most from additional financial support. We therefore recommend that the ONS, in addition to their current publications of infant mortality statistics, also report consistent infant mortality estimates by excluding births at <24 weeks and monitor trends in births and deaths occurring at <24 weeks. Consistency over time in reporting of infant mortality estimates is crucial in order to examine the impact of, for example, the introduction of universal credit starting in 2013, on early-life survival in England.

Socio-economic inequalities in infant mortality rates remained constant over time. The differences in mortality rates were smallest for perinatal deaths, which cover stillbirths and 55% of infant deaths, and largest for postneonatal deaths. Understanding the underlying causes of socio-economic inequalities in infant mortality is complex. A number of interrelated risk factors act from preconception, through pregnancy and after birth. These include maternal factors (maternal physical health prior to and during pregnancy, young or old age, smoking, substance misuse and nutritional intake during pregnancy),^28–31^ which in turn determine infant factors (including prematurity, low birthweight and congenital anomalies),^2^^,^^31^^,^^32^ all of which are influenced by socio-economic factors (parental occupation, income, education level and housing quality).^33–36^ We showed that accounting for differences in the distribution of gestational age by IMD quintile substantially reduced the risk of perinatal death (to a 26% higher risk in the most deprived compared to the least deprived IMD quintile). This suggests that inequalities in perinatal mortality could be reduced by preventing preterm birth. Therefore, as we have shown previously,^2^ policies focusing on improving maternal health and wellbeing before and during pregnancy to reduce preterm birth would lead to the largest reductions in inequalities in infant mortality.

The risk of postneonatal death (accounting for 29% of infant deaths) was 2.4 times higher in the most deprived IMD quintile compared to the least deprived and remained twice as high even after accounting for gestational age. Using more detailed data from our previous study, we found that these inequalities remained after further adjustment for congenital anomalies and birth weight (analyses not shown).^2^ This suggests that these inequalities are driven by other factors in the family and wider environment. According to the ONS, 28% of postneonatal deaths in 2006–2016 were unexpected or unexplained (including sudden infant death syndrome).^37^ These deaths could potentially be prevented through better support for families after birth, including support for postnatal mental health (enabling reductions in risk-taking behaviours: smoking, drinking and illicit drug use) and improved housing (to allow sufficient space for safe sleeping).

### Limitations of this study

We used national data from civil registration systems, which reduced selection bias and ensured that our results are generalizable to England. Since the ONS routinely link birth registration, death registration and NHS birth notifications, we were able to undertake a cohort study to examine mortality among children who were born in a particular year, rather than calculating crude rates as the number of deaths over the number of births in a year. By using aggregate data, we reduced the risk of individual disclosure and avoided the lengthy processes for ethics and data provider approvals.^38^ The data are now openly available on the ONS website. Conversely, because we used openly available aggregate data, we could only adjust for one risk factor, gestational age. We have previously shown that the contribution of other key risk factors, including birth weight and congenital anomalies, explains some of the differences in infant mortality between the deprivation quintiles.^2^ In addition, upstream maternal risk factors, including long-term conditions, smoking and body-mass index (BMI), are not currently recorded on either the ONS birth certificate or on the NHS birth notification data. The new Maternity Statistics Dataset which is currently being rolled out across England contains data on maternal BMI and smoking.^39^ Future linkage of this dataset to birth and death registrations and Hospital Episode Statistics data will allow further evaluation of the contribution of these factors to the socio-economic disparities in early-life survival.

Further, we were not able to exclude late terminations of pregnancy (TOP) from the counts of stillbirths, which may have a different aetiology to other stillbirths at early gestations. While 99.9% TOPs are carried out before 24 gestational weeks in England and Wales, later TOPs are allowed in some circumstances (such as risk to the mother’s life). In 2016, there were 226 TOPs at ≥24 weeks of gestation in England and Wales, and 225 were due to detection of foetal abnormalities.^40^

In addition, we used an area-level, rather than a household-level, indicator of socio-economic status, which likely underestimated observed socio-economic inequalities. The ONS holds information on parental occupation from birth records to derive an individual-level indicator based on the socio-economic classification of occupations. This information, however, is coded only for 10% of live births, and the classification has changed over time. Therefore, our analyses focussed on IMD, as this indicator was complete for all children and was reported consistently over time.^14^

The increase in infant mortality in England since 2014 can be accounted for by an increase in deaths among babies born at <24 weeks of gestation. The ONS should publish data on infant mortality based on births at ≥24 weeks of gestation and the proportion of births and deaths in children born at <24 weeks of gestation. Investments in maternal physical and mental health before and during pregnancy can be expected to substantially reduce socio-economic disparities in perinatal mortality in England. Finally, improved financial support and mental health services for families with children aged <1 year would also reduce socio-economic differences in postneonatal mortality.

## Supplementary Material

Online_Supplementary_Materials_ONS_paper_revised_Jan_2020_clean_fdaa025

## Data Availability

Data relevant to the current analysis are publically available and can be found at: https://www.ons.gov.uk/peoplepopulationandcommunity/birthsdeathsandmarriages/deaths/adhocs/010294numberoflivebirthsstillbirthsandinfantdeathsbyindexofmultipledeprivationimdandgestationalageatbirthforengland2006to2016.

## Notes

**Selina Nath,** Research Fellow in Epidemiology

**Pia Hardelid,** Associate Professor in Epidemiology

**Ania Zylbersztejn,** Research Fellow in Epidemiology
