# Supplementary material for: Are infant mortality rates increasing in England? The effect of extreme prematurity and early neonatal deaths
Source: J Public Health (Oxf). 2020 Mar 2;43(3):541–50. doi: 10.1093/pubmed/fdaa025 (PMC8458015; doi:10.1093/pubmed/fdaa025)
Supplement: Online_Supplementary_Materials_ONS_paper_revised_Jan_2020_clean_fdaa025 [file online_supplementary_materials_ons_paper_revised_jan_2020_clean_fdaa025.docx]

# Online Supplement – S1 further description of methods

**Definitions of infant and perinatal mortality**

Our primary outcome of interest was the *infant mortality* *rate per 1000 live births* calculated using Office for National Statistics (ONS) definition used for publication of national statistics:^1^

$$Infant mortality rate=\frac{Deaths aged 0-364 days}{Live Births}\times1000$$

Infant mortality rate was broken down by age at death as:

Early neonatal (0-6 days) mortality rate per 1000 live births: $\frac{Deaths aged 0-6 days}{Live Births}\times1000$

Late neonatal (7-27 days) mortality rate per 1000 live births: $\frac{Deaths aged 7-27 days}{Live Births}\times1000$

Post-neonatal (28-364 days) mortality rate per 1000 live births: $\frac{Deaths aged 28-364 days}{Live Births}\times1000$

Our secondary outcomes were the *stillbirth rate per 1000 total births* and the *perinatal mortality rate per 1000 total births* defined as:

Stillbirth rate per 1000 total births: $\frac{Stillbirths}{Live Births+Stillbirths}\times1000$

Perinatal mortality rate per 1000 total births: $\frac{Stillbirths+Deaths aged 0-6 days}{Live Births+Stillbirths}\times1000$

**Description of Interrupted Time-Series Analysis (ITSA)**

We used Interrupted Time-Series Analysis (ITSA) to estimate trends in mortality rates before and after 2014 and to assess the magnitude of change.^2^ Specifically, we used a linear regression model with mortality rate as outcome, adjusted for year of birth (as a continuous variable), a binary indicator of the period before and after 2014 (equal to 0 in 2006-2013, and 1 afterwards), and an effect modification term between the period indicator and year of birth as a measure of change in trend in 2014. The model looked as follows:

$$Y_{t}=\beta_{0}+\beta_{1}T_{t}+\beta_{2}X_{t}+\beta_{3}T_{t}X_{t}$$

Where $Y_{t}$ is the mortality rate, $T_{t}$ is number of years since 2006, $X_{t}$ is the binary indicator of period before and after 2014, and $T_{t}X_{t}$ is the interaction term. Therefore, $\beta_{0}$ described mortality rate at baseline (in 2006), $\beta_{1}$described annual rate of change since 2006, $\beta_{2}$ is the change in expected and observed mortality rate in 2014, and $\beta_{3}$ is the change in slope of the trend in mortality rate before and after 2014. We used estimate of $\beta_{1}$ to describe trends before 2014, and $\beta_{1}+\beta_{3}$ to derive the trend in mortality after 2014.

Online Supplement – S2 additional results

**Supplementary table S1**: Change in the slope of fitted trend in infant mortality per 1000 births* by age-at-death before and after 2014 from interrupted time-series analysis

|  | **All births and deaths** | | **Births and deaths with gestational age ≥24 weeks** | |
| --- | --- | --- | --- | --- |
| **Age at death:** | **before 2014** | **after 2014** | **before 2014** | **after 2014** |
| **Infancy  (0-364 days)** | -0.15  (-0.17, -0.13) | 0.07  (0.04, 0.11) | -0.14  (-0.17, -0.11) | -0.04 (-0.05, -0.03) |
| **Early neonatal (0-6 days)** | -0.08  (-0.09, -0.07) | 0.09  (0.07, 0.11) | -0.07  (-0.08, -0.05) | -0.02  (-0.03, -0.01) |
| **Late neonatal (7-27 days)** | -0.02  (-0.04, -0.01) | 0.001  (-0.002, 0.004) | -0.02  (-0.04, -0.01) | -0.001  (-0.008, 0.006) |
| **Post neonatal (28-364 days)** | -0.05  (-0.08, -0.02) | -0.02 (-0.02, -0.01) | -0.05  (-0.08, 0.02) | -0.02  (-0.02, -0.01) |
| **Perinatal (stillbirths & early neonatal deaths)** | -0.15  (-0.21, -0.09) | -0.04  (-0.10, 0.03) | -0.14  (-0.20, -0.07) | -0.14  (-0.16, -0.12) |
| **Stillbirths** | -0.07  (-0.13, -0.01) | -0.13  (-0.16, -0.09) | -0.07 (-0.13, -0.01) | -0.12  (-0.15, -0.09) |

*note that perinatal mortality and stillbirth rates are calculated per 1000 total births. All other rates are calculated per 1000 live births

**Supplementary table S2**: Crude mortality rates per 1000 live births, or 1000 total births (stillbirth & perinatal mortality) with 95% confidence intervals

|  | Infant mortality | Early neonatal mortality | Late neonatal mortality | Post neonatal mortality | Stillbirth rate | Perinatal mortality |
| --- | --- | --- | --- | --- | --- | --- |
| Year of birth |  |  |  |  |  |  |
| 2006 | 4.78 (4.61, 4.95) | 2.59 (2.47, 2.72) | 0.82 (0.75, 0.89) | 1.36 (1.27, 1.45) | 5.36 (5.18, 5.54) | 7.94 (7.72, 8.16) |
| 2007 | 4.71 (4.54, 4.87) | 2.50 (2.38, 2.62) | 0.71 (0.65, 0.78) | 1.49 (1.40, 1.59) | 5.10 (4.92, 5.27) | 7.58 (7.37, 7.79) |
| 2008 | 4.52 (4.36, 4.68) | 2.40 (2.28, 2.52) | 0.72 (0.66, 0.79) | 1.40 (1.31, 1.48) | 5.00 (4.83, 5.17) | 7.39 (7.18, 7.59) |
| 2009 | 4.33 (4.17, 4.49) | 2.31 (2.19, 2.42) | 0.71 (0.64, 0.77) | 1.32 (1.23, 1.40) | 5.09 (4.92, 5.26) | 7.39 (7.18, 7.59) |
| 2010 | 4.17 (4.02, 4.32) | 2.25 (2.14, 2.36) | 0.64 (0.58, 0.70) | 1.28 (1.20, 1.37) | 5.04 (4.87, 5.21) | 7.28 (7.07, 7.48) |
| 2011 | 4.14 (3.99, 4.29) | 2.25 (2.14, 2.36) | 0.69 (0.63, 0.75) | 1.20 (1.12, 1.28) | 5.20 (5.03, 5.37) | 7.44 (7.23, 7.64) |
| 2012 | 3.87 (3.72, 4.01) | 2.10 (1.99, 2.20) | 0.62 (0.56, 0.68) | 1.15 (1.07, 1.23) | 4.80 (4.64, 4.96) | 6.88 (6.69, 7.08) |
| 2013 | 3.77 (3.62, 3.92) | 2.05 (1.94, 2.15) | 0.65 (0.59, 0.71) | 1.07 (0.99, 1.15) | 4.60 (4.44, 4.76) | 6.63 (6.44, 6.83) |
| 2014 | 3.54 (3.40, 3.68) | 1.96 (1.86, 2.07) | 0.56 (0.50, 0.61) | 1.02 (0.94, 1.10) | 4.55 (4.39, 4.72) | 6.51 (6.31, 6.70) |
| 2015 | 3.57 (3.42, 3.71) | 2.02 (1.91, 2.13) | 0.55 (0.50, 0.61) | 1.00 (0.92, 1.07) | 4.37 (4.21, 4.53) | 6.38 (6.19, 6.58) |
| 2016 | 3.69 (3.54, 3.83) | 2.14 (2.03, 2.26) | 0.56 (0.50, 0.61) | 0.99 (0.91, 1.06) | 4.30 (4.14, 4.46) | 6.44 (6.24, 6.63) |
| IMD quintile |  |  |  |  |  |  |
| Q1: Most deprived | 5.51 (5.41, 5.61) | 2.86 (2.79, 2.94) | 0.88 (0.84, 0.92) | 1.77 (1.71, 1.83) | 6.03 (5.92, 6.14) | 7.80 (7.68, 7.92) |
| Q2 | 4.34 (4.25, 4.45) | 2.34 (2.27, 2.42) | 0.69 (0.65, 0.74) | 1.31 (1.25, 1.36) | 5.10 (4.99, 5.21) | 6.55 (6.43, 6.67) |
| Q3 | 3.56 (3.46, 3.66) | 2.00 (1.93, 2.08) | 0.57 (0.53, 0.62) | 0.98 (0.93, 1.03) | 4.56 (4.45, 4.67) | 5.84 (5.71, 5.97) |
| Q4 | 3.15 (3.05, 3.25) | 1.80 (1.72, 1.88) | 0.52 (0.48, 0.56) | 0.83 (0.78, 0.88) | 4.04 (3.93, 4.15) | 5.22 (5.09, 5.35) |
| Q5: Least deprived | 2.89 (2.79, 2.99) | 1.70 (1.62, 1.77) | 0.46 (0.42, 0.50) | 0.74 (0.69, 0.79) | 3.68 (3.57, 3.79) | 4.75 (4.63, 4.88) |
| Gestational age (week) |  |  |  |  |  |  |
| < 24 | 870 (862, 878) | 791 (782, 800) | 45.7 (41.1, 50.6) | 33.2 (29.3, 37.5) |  |  |
| 24 - 27 | 240 (235, 245) | 127 (123, 131) | 56.3 (53.5, 59.2) | 57.0 (54.1, 59.9) | 265 (260, 269) | 358 (353, 363) |
| 28 - 31 | 45.4 (43.8, 47.1) | 24.4 (23.2, 25.7) | 9.37 (8.61, 10.2) | 11.6 (10.8, 12.5) | 91.1 (88.9, 93.3) | 113 (111, 116) |
| 32 - 36 | 8.70 (8.43, 8.97) | 4.02 (3.84, 4.21) | 1.51 (1.40, 1.63) | 3.16 (3.00, 3.33) | 19.1 (18.7, 19.5) | 23.0 (22.6, 23.5) |
| 37 - 41 | 1.50 (1.47, 1.53) | 0.49 (0.48, 0.51) | 0.26 (0.25, 0.28) | 0.75 (0.73, 0.77) | 1.78 (1.75, 1.81) | 2.27 (2.24, 2.31) |
| 42 + | 1.33 (1.19, 1.47) | 0.64 (0.54, 0.74) | 0.18 (0.13, 0.24) | 0.51 (0.43, 0.60) | 1.33 (1.20, 1.48) | 1.97 (1.80, 2.14) |
| Missing | 13.1 (12.1, 14.1) | 10.4 (9.53, 11.3) | 0.99 (0.74, 1.31) | 1.71 (1.37, 2.11) | 2.30 (1.90, 2.76) | 12.7 (11.7, 13.7) |

IMD= index of multiple deprivation.

**Supplementary table S3**: Unadjusted and adjusted risk ratios (95% confidence intervals) from log-binomial models for early neonatal mortality and stillbirth rates by year of birth and IMD quintile in England, adjusted for gestational age.

|  | Early neonatal  Death at 0 – 6 days | | Stillbirths** | |
| --- | --- | --- | --- | --- |
|  | **Model 1** | **Model 2** | **Model 1** | **Model 2** |
| Year of birth* | 0.96  (0.95 - 0.96) | 0.96  (0.96 – 0.97) | 0.98  (0.98 - 0.98) | 0.98  (0.98 - 0.99) |
| IMD quintile |  |  |  |  |
| Q1: Most deprived | 1.64  (1.53 - 1.75) | 1.26  (1.18 - 1.35) | 1.65  (1.59 - 1.71) | 1.28  (1.24 - 1.32) |
| Q2 | 1.35  (1.26 - 1.45) | 1.14  (1.07 - 1.22) | 1.39  (1.34 - 1.44) | 1.18  (1.14 - 1.22) |
| Q3 | 1.20  (1.11 - 1.29) | 1.10  (1.02 - 1.18) | 1.24  (1.19 - 1.29) | 1.14  (1.10 - 1.18) |
| Q4 | 1.11  (1.03 - 1.20) | 1.04  (0.96 - 1.12) | 1.10  (1.05 - 1.15) | 1.04  (1.00 - 1.08) |
| Q5: Least deprived | Reference | Reference | Reference | Reference |
| Gestational age (weeks) |  |  |  |  |
| 24 - 27 |  | 250.78  (239.14 - 262.98) |  | 145.42  (141.77 - 149.16) |
| 28 - 31 |  | 48.69  (45.79 - 51.78) |  | 50.43  (48.93 - 51.98) |
| 32 - 36 |  | 8.10  (7.65 - 8.58) |  | 10.66  (10.37 - 10.96) |
| 37 – 41 |  | Reference |  | Reference |
| 42 or over |  | 1.27  (1.08 - 0.48) |  | 0.75  (0.67 - 0.83) |

IMD = Index of Multiple Deprivation.
*year of birth has been centred at 2006
**Stillbirth rates are based on total births.
Model 1 included year of births and IMD quintile. Model 2 was further adjusted for gestational age.

**Supplementary table S4**: Proportions of births and deaths registered at <24 weeks’ gestation and with missing gestational age.

|  | Live births | | Early neonatal deaths (0-6 days) | |
| --- | --- | --- | --- | --- |
| Year of birth | Gestational age <24 weeks* | Missing gestational age** | Gestational age <24 weeks* | Missing gestational age** |
| 2006 | 0.11% | 0.70% | 35.2% | 3.16% |
| 2007 | 0.09% | 1.08% | 30.5% | 5.44% |
| 2008 | 0.11% | 1.06% | 36.6% | 3.97% |
| 2009 | 0.10% | 1.04% | 33.4% | 3.74% |
| 2010 | 0.11% | 1.01% | 38.1% | 2.22% |
| 2011 | 0.10% | 0.68% | 37.1% | 2.34% |
| 2012 | 0.10% | 0.39% | 40.1% | 1.99% |
| 2013 | 0.10% | 0.31% | 40.1% | 2.58% |
| 2014 | 0.10% | 0.48% | 40.6% | 2.70% |
| 2015 | 0.11% | 0.48% | 42.1% | 3.21% |
| 2016 | 0.12% | 0.29% | 46.8% | 3.38% |

*based on births and deaths with complete gestational age
**based on all births

**Supplementary table S5**: Number (%) of live births and early neonatal deaths (at 0-6 days) in infants born <24 weeks gestation and early neonatal mortality per 1000 live births at <24 weeks in the most deprived and least deprived quintile of socioeconomic status.

| Year of birth | Live births at <24 weeks (number, % of all) | | Early neonatal deaths  (number, % of all) | | Early neonatal mortality per 1000 live births at <24 weeks (95% CI) | |
| --- | --- | --- | --- | --- | --- | --- |
|  | Q1: Most deprived | Q5: Least deprived | Q1: Most deprived | Q5: Least deprived | Q1: Most deprived | Q5: Least deprived |
| 2006 | 260 (0.15%) | 79 (0.08%) | 197 (35%) | 62 (32%) | 758 (701, 808) | 785 (678, 869) |
| 2007 | 205 (0.12%) | 81 (0.08%) | 164 (32%) | 64 (36%) | 800 (739, 852) | 790 (685, 873) |
| 2008 | 287 (0.16%) | 60 (0.06%) | 227 (40%) | 45 (29%) | 791 (739, 837) | 750 (621, 853) |
| 2009 | 243 (0.13%) | 61 (0.06%) | 188 (38%) | 50 (25%) | 774 (716, 825) | 820 (700, 906) |
| 2010 | 246 (0.14%) | 70 (0.07%) | 195 (40%) | 58 (38%) | 793 (737, 842) | 829 (720, 908) |
| 2011 | 249 (0.14%) | 74 (0.07%) | 198 (38%) | 65 (38%) | 795 (740, 844) | 878 (782, 943) |
| 2012 | 258 (0.14%) | 77 (0.08%) | 209 (42%) | 71 (39%) | 810 (757, 856) | 922 (838, 971) |
| 2013 | 241 (0.13%) | 84 (0.09%) | 195 (42%) | 72 (46%) | 809 (754, 857) | 857 (764, 924) |
| 2014 | 243 (0.14%) | 79 (0.08%) | 184 (40%) | 56 (39%) | 757 (698, 810) | 709 (596, 806) |
| 2015 | 249 (0.14%) | 79 (0.08%) | 196 (41%) | 60 (44%) | 787 (731, 836) | 759 (650, 849) |
| 2016 | 267 (0.15%) | 96 (0.10%) | 206 (45%) | 80 (50%) | 772 (716, 821) | 833 (744, 902) |

All calculations are based on births and deaths with complete gestational age; CI=confidence interval

**Supplementary table S6**: Mortality rates per 1000 live births (per 1000 total births for stillbirth rate) with and without births with missing gestational age

|  | Infancy  (0-364 days) | Early neonatal (0-6 days) | Late neonatal (7-27 days) | Post neonatal (28-364 days) | Stillbirths* |
| --- | --- | --- | --- | --- | --- |
| Based on all births and deaths | 4.09 | 2.23 | 0.66 | 1.21 | 4.85 |
| Based on births and deaths with complete gestational age | 4.03 | 2.18 | 0.65 | 1.20 | 4.87 |

**Supplementary figure S7**: Early neonatal mortality (at 0-6 days) rates per 1000 live births calculated based on 1) all births 2) excluding births with missing gestational age 3) excluding births at <24 weeks’ gestation and 4) excluding both births with missing gestational age and at <24 weeks’ gestation.

**References**

1. Office for National Statistics. User guide to child and infant mortality statistics 2019 [Available from: <https://www.ons.gov.uk/peoplepopulationandcommunity/birthsdeathsandmarriages/deaths/methodologies/userguidetochildmortalitystatistics> accessed 23/09/2019.

2. Linden A. Conducting interrupted time-series analysis for single-and multiple-group comparisons. *The Stata Journal* 2015;15(2):480-500.
